# Resolution of Abfraction-Associated Gingival Fenestration Utilizing Connective Tissue Grafting

**DOI:** 10.1155/2019/6810670

**Published:** 2019-06-12

**Authors:** Braydon Haskell, J. Kobi Stern, Jordan Ghiassi, Andrew Kurialacherry, Sadja Gaud-Quintana, Mark E. Peacock

**Affiliations:** The Dental College of Georgia at Augusta University, Augusta, GA, USA

## Abstract

**Introduction:**

Gingival fenestration (GF) is a distinct clinical entity of uncertain etiology that is seldom documented in the literature. It has been associated mainly with submucosal mechanical irritants such as calculus that subsequently create an opening in the oral soft tissue, usually at facial anterior sites. Surgical correction may be indicated to address functional and/or esthetic concerns.

**Case Presentation:**

The patient, a 74-year-old male, presented to the clinic with a chief complaint of “something is poking through my gum.” Clinical exam revealed a gingival fenestration on the facial of tooth #11, associated with what appeared to be a pronounced noncarious cervical lesion (NCCL). Surgical treatment consisted of a connective tissue graft and odontoplasty of the sharp protruding edge of the root surface. Healing was uneventful with excellent closure of the fenestration and no evidence of recurrence after 18 months of follow-up.

**Conclusion:**

GF is a perforation of the mucosa typically associated with underlying sharp mechanical etiology. This report describes a fenestration that developed from a probable abfractive lesion, which later was successfully closed and exhibits long-term stability.

## 1. Introduction

Gingival fenestration is an opening through oral keratinized mucosa that is reported infrequently, possibly due to lack of symptoms and/or patient unawareness [1]. Unlike fenestrations, the gingival margin for a dehiscence is noncontinuous and apically positioned. Although previously mentioned in the literature, Lane in 1977 was the first to describe it as a distinct pathologic entity [2]. The etiological basis for GF has not been completely elucidated but is probably multifactorial.

Local factors that have been reported to be associated with GF include plaque/calculus [1–4], cervical enamel projections (CEP) [5], tooth malpositioning [1, 2], trauma [3], chewing habits/foreign body (gutka) [6], occlusal factors [7], and implant fixture threads [8]. Anatomic factors associated with noncarious cervical lesions (abfraction, abrasion) could possibly lead to sharp irregular cervical “notching,” [9] resulting in a soft tissue fenestration [10]. Almost all reported cases of GF occurred on the facial surfaces of incisor teeth.

Soft tissue fenestrations linked with NCCLs are almost never documented. NCCLs may have very rough and serrated line angles that could lead to GF. A strong relationship between NCCLs and occlusal overload (bruxism, grinding) has been reported [11], and patients with group function can have NCCLs occurring six times more frequently than those with cuspid-protected occlusion [12]. The theoretical concept of abfraction, described by Lee and Eakle [13], is controversial, but this phenomenon is still supported by a multitude of practitioners.

Most gingival fenestrations described have occurred on the facial surfaces of thin unattached gingiva and are associated with heavy calculus deposits. This case report documents a GF linked to a subgingival NCCL, along with its surgical treatment and follow-up.

## 2. Case Report

**Figure 1:**
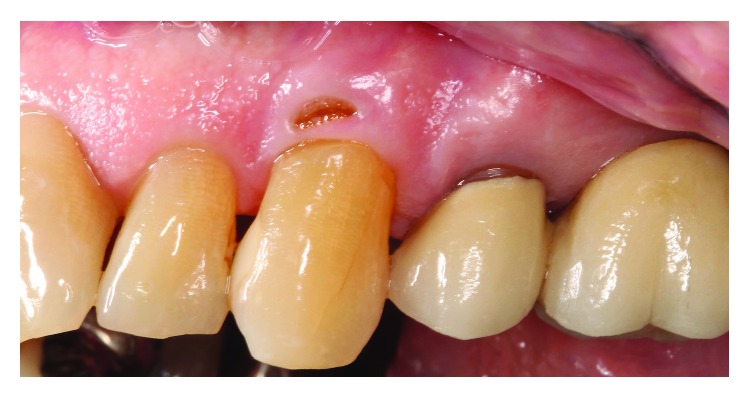
Fenestration on facial gingiva #11.

**Figure 2:**
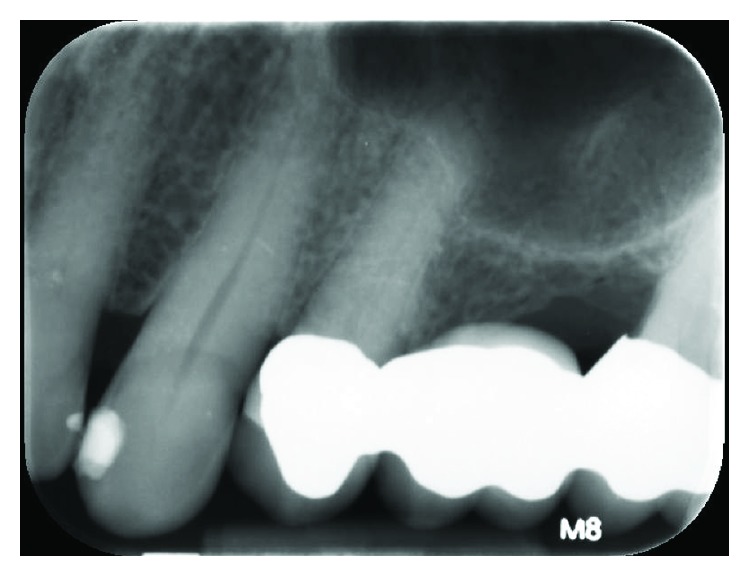
Periapical radiograph #11.

A 74-year-old Caucasian male was referred to the Periodontics Clinic of the Dental College of Georgia, Augusta University, Augusta, Georgia, in May of 2015, with a chief complaint of “something has poked through my gum.” The patient had been on a routine three-month maintenance cycle and reported no previous incidence of the condition, which had been present approximately six months. The medical history review was remarkable for hypertension, hyperlipidemia, and implanted artificial pacemaker and noncontributory social history (no use of gutka products). Current medications included metoprolol, amlodipine, losartan, ezetimibe/simvastatin, and aspirin daily. Clinical examination revealed a 3 × 5 *mm* fenestration through the facial gingiva at tooth #11 (Figure 1). Patient reported daily brushing and flossing using an extra soft toothbrush that was routinely changed at each hygiene appointment. Oral hygiene instructions were reinforced and reviewed at each appointment, and through demonstration, the patient exhibited proper brushing and flossing technique. All gingival probings were ≤3 mm with no mobility, no suppuration or swelling present, and no decay detected, but there was a sharp, pointy edge of what appeared to be the coronal border of a NCCL (abfraction). Evidence of previous occlusal trauma to #11 was visible with blunted cusp tip and craze line; similarly, tooth #10 displayed a wear facet. The patient does not present with cuspid-protected occlusion. The tooth tested vital, and the lesion was asymptomatic, except for the noticeable perforation of the gingiva. A radiograph taken showed only mild interproximal horizontal bone loss (Figure 2).

Various treatment options were discussed with the patient including no treatment, gingivectomy and direct restoration, gingival flap and odontoplasty, or connective tissue grafting (CTG) with odontoplasty, along with limited occlusal adjustment. The patient was thoroughly informed of all aspects of the treatment for this unique association between NCCL and GF. CTG was preferred to improve upon the patient's thin phenotype in order to address the patient's chief complaint. He was counseled and consented to surgical treatment to correct the soft tissue defect and odontoplasty of the root surface irregularity.

**Figure 3:**
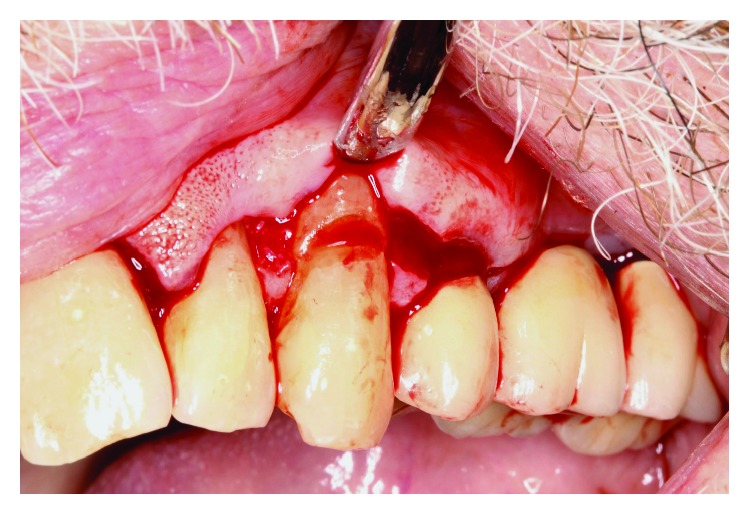
Prominent edge of NCCL #11.

**Figure 4:**
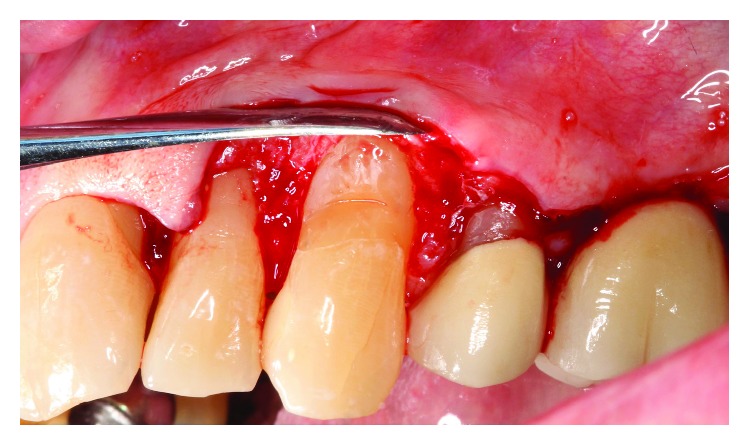
Postodontoplasty #11.

**Figure 5:**
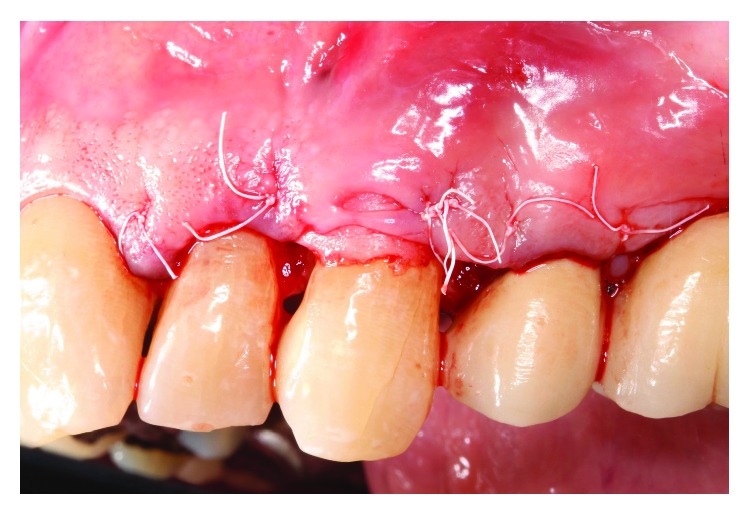
Closure of surgical graft site.

Occlusal adjustment was made on #11 cuspid until light occlusal contacts evenly distributed bilaterally. Under local anesthesia with 2% lidocaine and 1 : 100,00 epinephrine, a facial sulcular-modified papilla preservation incision was made from #10 to #12, and a mucoperiosteal flap reflected revealing a NCCL with prominent sharp edges at #11 (Figure 3). The root prominence of the NCCL was reduced with high speed rotary instrumentation (Figure 4), and the site prepared for soft tissue grafting. A connective tissue graft was taken from the left maxillary tuberosity region, and closure was obtained with 5-0 PTFE monofilament sutures after copious irrigation of the surgical site (Figure 5).

**Figure 6:**
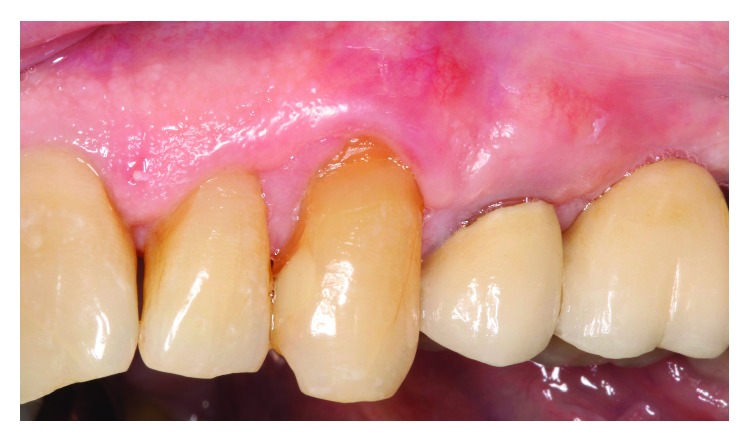
2-month postop healing.

**Figure 7:**
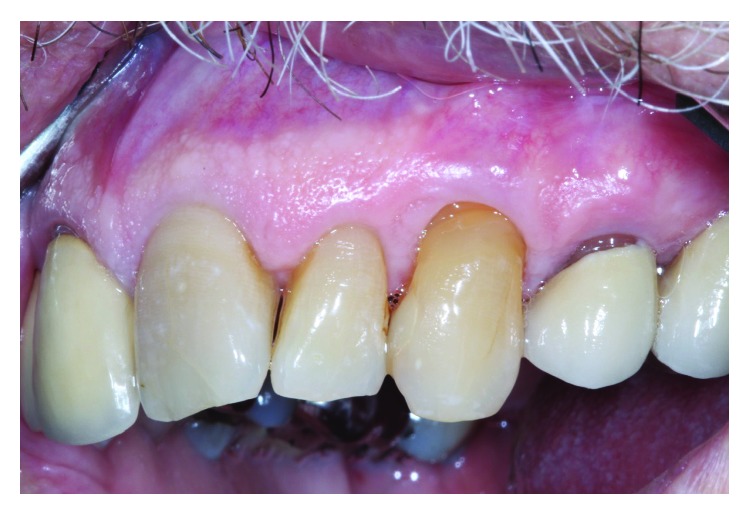
18-month follow-up.

The surgical site healed without complication.  Figure 6 shows healing at 2 months with correction of the gingival perforation evident. An 18-month follow-up (Figure 7) reveals mature healing of the defect, and the patient has had no complaints. Noticeable from the 2-month to the 18-month postop is approximately 1 mm coronal gingival migration by “creeping attachment,” a phenomenon described by Goldman and Cohen [14].

## 3. Discussion

Documented gingival fenestration lesions are uncommon, though the prevalence could be higher due to underreporting. The pathogenesis of GF is uncertain; however, it has been proposed that it is a transient stage in gingival cleft formation from a combination of thin tissue and subgingival irritants [2]. Because these lesions are often asymptomatic, the perceived need for treatment to many patients is minimal.

Most scientific reports reveal calculus to be the most common irritant that results in a soft tissue fenestration. Davies et al.'s review on management of NCCLs is the only other documentation in the literature describing a cervical cavity (abfraction) causing a GF [10]. They suggest that because the overlying gingiva exerted a “protective” effect, the lesion etiology was abfraction without any abrasive or erosive element. As is in the case presented here, abrasion and/or erosion would probably have produced recession versus a protrusion through the gingiva.

Most documented accounts of GF occurred on the facial of anterior teeth (maxillary and mandibular). Comparably, almost all NCCLs also occur on the facial, with only 2% of them reported to exist on the lingual or palatal surface [15]. Excessive occlusal forces producing stress near the cementoenamel junction (CEJ), combined with mechanical abrasion, may predispose a patient to gingival recession. However, as can be seen in this case, a fenestration in the gingiva may occasionally form which will not revert to a recession defect. This rare defect was successfully closed by surgical treatment, with long-term verified follow-up.

Surgical rationale for utilizing an autogenous CTG versus acellular dermal matrix stems from historic success as the gold standard for long-term stability, tissue thickening, and ability to remain exposed [16]. Access to the lesion was a critical component to the surgical intervention; thus, reflection of a mucoperiosteal flap provided adequate visual and surgical field to perform odontoplasty.

## 4. Conclusion

Gingival fenestration is a perforation of the mucosa typically associated with underlying sharp mechanical etiology. This report describes a fenestration that developed from a probable abfractive lesion, which later was successfully closed and exhibits long-term stability. Further research and follow-up would provide more evidence to support the results of this case report.

## Additional Points

*Summary*. This case documents an infrequently reported soft tissue lesion, the gingival fenestration, and the accompanying probable etiology, a prominent subgingival abfraction.
